# Association between serum uric acid level and bone mineral density in men more than 50 years of age

**DOI:** 10.3389/fendo.2023.1259077

**Published:** 2023-11-30

**Authors:** Sujin Kim, Seulki Lee, Hyuktae Kwon

**Affiliations:** ^1^ Department of Family Medicine, Seoul National University Hospital, Jongno-gu, Seoul, Republic of Korea; ^2^ Department of Family Medicine, Seoul National University College of Medicine, Daehak-ro-Jongno-gu, Seoul, Republic of Korea

**Keywords:** uric acid, bone mineral density, osteopenia and osteoporosis, fat free mass, skeletal muscle mass index

## Abstract

**Background:**

The results of previous studies on the association between serum uric acid levels and bone mineral density and the risk of osteoporosis are controversial. Fat free mass (FFM) is more strongly associated with bone mineral density (BMD) than it is with body fat mass (BFM). Skeletal muscle mass is assumed to contribute indirectly to the relationship between serum uric acid levels and BMD. Therefore, we aimed to evaluate the association between serum uric acid levels and BMD and abnormal BMD (at least osteopenia) by considering body composition in men aged ≥ 50 years.

**Methods:**

This was a retrospective observational cross-sectional study. We used data obtained from 2,991 men aged ≥50 years who completed questionnaires, anthropometric surveys, laboratory tests, and bone mineral density scans. A subgroup analysis of 1,135 men who additionally underwent body composition data analysis using Inbody^®^ was performed. Multiple linear regression analysis was used to explore the relationship between serum uric acid levels and BMD at three sites (L1-L4, Femur neck, Femur total). In addition, multiple logistic regression analysis was performed to determine the association of serum uric acid levels with abnormal BMD (at least osteopenia).

**Results:**

Positive correlations between serum uric acid levels and BMD at the three sites (L1-L4, Femur neck, Femur total) were observed in unadjusted and fully adjusted models except the BMD of the femoral neck (P-value=0.054).

Furthermore, FFM and skeletal muscle mass index (SMI) showed positive association with serum uric acid level and BMD at three sites, with statistical significance. An increase in serum uric acid level was associated with a lower risk of abnormal BMD after adjusting for confounders including FFM and SMI.

**Conclusion:**

Serum uric acid level was positively associated with BMD at three sites and had a protective effect against abnormal BMD after adjusting for multiple confounders, including FFM and SMI, in men aged ≥ 50 years.

## 1 Introduction

The prevalence of osteoporosis increases with age (1). As Korea is rapidly becoming a super-aged society, the prevalence of osteoporosis and osteoporotic fractures is increasing steeply, leading to significant social costs (2). Particularly, osteoporotic fractures resulting from bone fragility cause pain, prolonged immobilization, and even death (3). According to data from the National Health Insurance Service (NHIS) regarding osteoporotic patients in 2015, the death rate within 1 year after osteoporotic femur fracture was 21% in men and 14% in women, and osteoporotic lumbar fracture was 9% in men and 4% in women. Data of 66-year-old women who underwent life turning point health examination provided by the NHIS showed that fracture risk was higher among patients with osteopenia than those with normal bone mineral density (BMD) (4). According to the national nutritional survey data from 2008 to 2011, the prevalence of osteopenia in adults over 50 years of age was 47.9% (46.8% in men and 48.9% in women) (2) Therefore, for the prevention of future fractures, it is important to aggressively manage not only osteoporosis but also osteopenia (4).

Uric acid is recognized as a pro-oxidative marker intracellularly but also a protective factor as an anti-oxidant extracellularly (5, 6). Hyperuricemia has been recognized as a risk factor for not only gout and kidney stones, but also as a metabolic syndrome (5, 7, 8). In addition it is either a consequence or a cause of these related conditions (6). However, paradoxically, uric acid is responsible for up to two-third of total plasma antioxidant capacity by scavenging for peroxyl radicals (ROO-) and chelating iron (9, 10). Moreover, an imbalance between oxidative stress and antioxidation affects bone remodeling (5, 8). In a 2016 systematic review and meta-analysis, higher serum uric acid level was associated with higher BMD, lower prevalence of osteoporosis, and new fractures during the follow-up period (8). However, some studies reported no association (3, 11, 12) or different association by sex (13, 14). Besides, the majority of studies have been on women.

In addition, high body weight and BMI have long been demonstrated to have a protective effect against the decline in BMD (15). Based on a meta-analysis of 44 studies, Ho-Pham et al. showed that fat free mass (FFM) was more associated with BMD than did body fat mass (BFM) in men and women (16). Dong XW et al. found that serum uric acid level had positive correlations with BMD and skeletal muscle mass index (SMI). Moreover, path analysis explained that beneficial association between serum uric acid and BMD might be mediated by increased SMI (17). Furthermore, although previous studies on the relationship between serum uric acid levels and skeletal muscle mass are limited and controversial, a positive correlation is estimated among relatively healthy, middle-aged and older adults (18, 19). Therefore, we aimed to evaluate the relationship between serum uric acid and BMD, considering body composition in men aged ≥50 years.

## 2 Methods

### 2.1 Study participants

**Figure 1:**
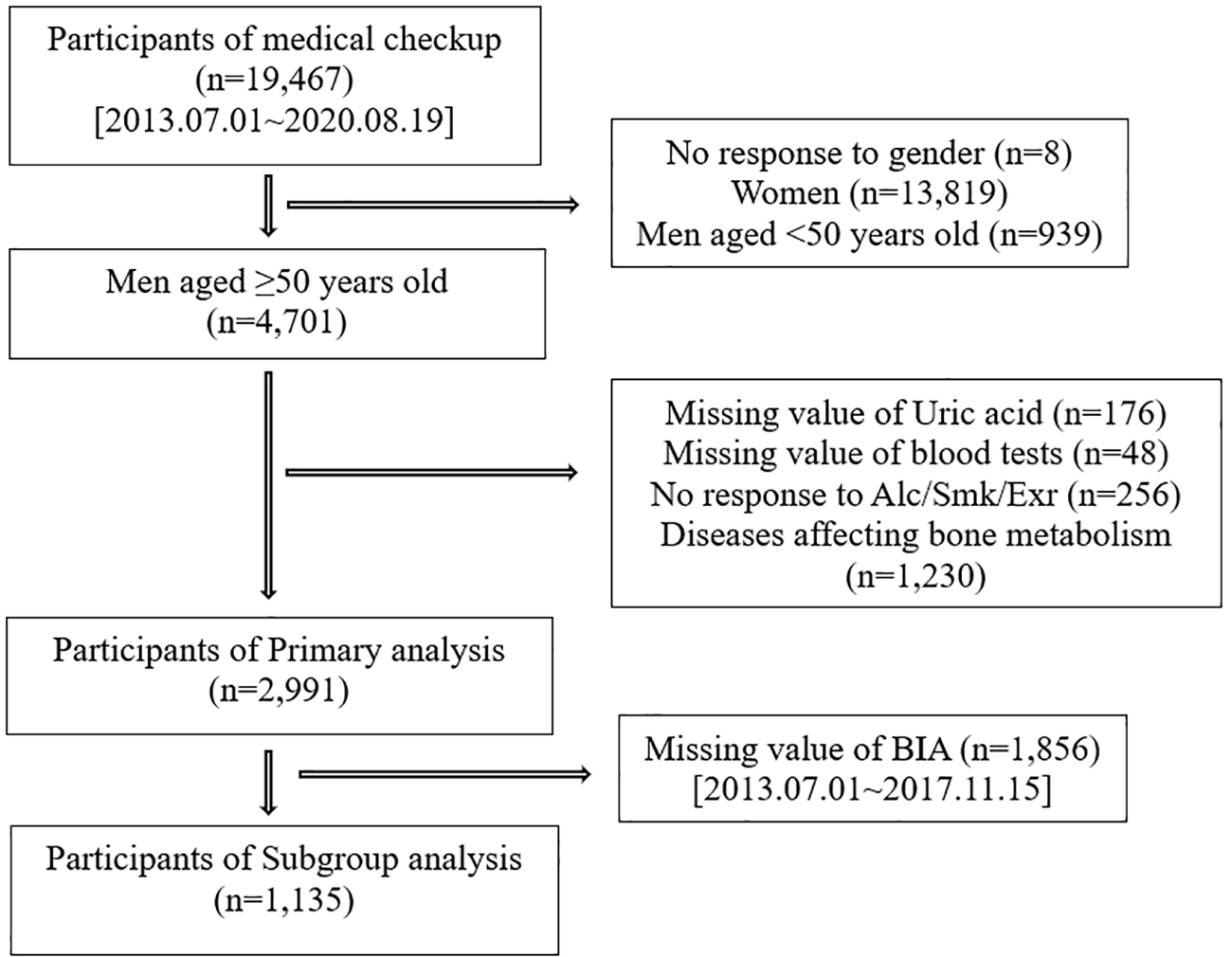
Flow chart of participants.

We investigated participants who voluntarily visited the Health Promotion/Disease Prevention Center of Seoul National University Hospital, Seoul, Korea between July 1, 2013 and August 19, 2020, for a health-screening program. The study population consisted of 19,467 participants who completed questionnaires, anthropometric surveys, laboratory tests, and BMD scans. We excluded 14,766 participants for the following reasons: no response to the question on sex (n=8), women (n=13,819), and men aged <50 years (n=939). Further exclusion criteria were as follows: missing uric acid values (n=176), missing laboratory research variables (n= 48), no response to any questions on smoking (20), alcohol (21) and exercise (22) (n=256), and medical histories affecting bone metabolism, such as osteoporosis, gout, chronic kidney disease, hyperthyroidism (23), chronic liver disease (24, 25), cancer, history of transplantation (26), rheumatic disease, autoimmune disease (27–29), and skeletomuscular disease (n=1,230). Therefore, 2,991 participants were included in the primary analysis. Afterward, we analyzed the data of 1,135 participants with body composition data to investigate the association of serum uric acid with BMD, considering FFM and SMI (**Figure 1**).

### 2.2 Bone mineral density test

BMD was evaluated using dual-energy X-ray absorptiometry (Model; BHR-3-76, USA), and the BMD and T score of three sites (lumbar spine 1-4, the femoral neck and femur total) were extracted. Using the minimum T score, osteoporosis was defined as a T score of 2.5 SD below the young adult level (T score ≤ -2.5), osteopenia was defined as a T score of 1-2.5 SD below the young adult level (-2.5 < T score < -1) and abnormal BMD was defined as a T score <1 SD below the young adult level (T score < -1) (2, 30)

### 2.3 Anthropometric survey and body composition test

Anthropometric data were measured by trained personnel using standardized protocols and instruments. Height (m) and weight (kg) were measured using a digital scale and BMI (kg/m (2)) was calculated. Body composition test was performed with the Inbody 770 using bioelectrical impedance analysis (BIA); and we collected data on BFM (total weight of body fat, including surface and internal levels), FFM (weight of all tissues in the body except body fat), SMM (total weight of lean muscles present at every body segment), and SMI (appendicular skeletal muscle mass (kg)/height (2) (m) (2)) (31, 32).

### 2.4 Other clinical assessments

Medical history and lifestyle activities, including smoking, alcohol consumption, and exercise, were documented using self-reported questionnaires. For alcohol consumption, we surveyed the frequency per week and month and the amount of alcohol consumed at once. The participants were categorized as non-drinkers (never and past drinkers) and current drinkers. According to the Korean guidelines for adequate alcohol consumption, “adequate drinkers” was defined as < eight drinks per week in men aged < 65 years and < four drinks per week in men aged ≥ 65 years (21). Smoking status was classified as nonsmoker (never or past smoker) or current smoker. In addition, we also surveyed the frequency and duration by intensity (vigorous, intermediate, or low) of the exercise types. Adequate exercise was defined as > 150 min/week of intermediate intensity or > 75 min/week of vigorous intensity (22).

### 2.5 Laboratory measurements

Blood tests were performed via venipuncture after overnight fasting. Laboratory examinations included the determination of serum uric acid level as the main variable and other variables associated with bone metabolism, including alkaline phosphatase (ALP), OHVitD3, calcium (Ca), phosphorous (P), blood urea nitrogen (BUN), creatinine (Cr), high sensitivity C-reactive protein (hsCRP), albumin (Alb), total cholesterol (TC), triglycerides (TG), low density lipoprotein (LDL), high density lipoprotein (HDL), fasting blood sugar (FBS), and glycated hemoglobin (HbA1c). High hsCRP refers to hsCRP >0.5 (reference value: 0–0.5). Because TG, one of the confounders, does not follow a normal distribution owing to skewness and kurtosis, we applied a natural log. For the exclusion criteria, we checked free T4 (fT4), thyroid-stimulating hormone (TSH) for hyperthyroidism (23) and hepatitis B surface antigen (HBsAg), hepatitis C antibody (anti-HCV) for chronic liver disease, and hepatitis B or C carriers (24, 25). In addition, we calculated eGFR using the MDRD equation, and excluded eGFR<60ml/min/1.73m (2) for chronic kidney disease (CKD) (33).

### 2.6 Statistical analyses

Statistical analyses were performed using STATA version 16.0. The general characteristics of the participants grouped according to abnormal BMD were compared using the independent t-test for continuous variables and Pearson’s chi-square test for categorical variables. We performed multiple linear regression to examine the relationship between serum uric acid levels and BMD, and the following variables were corrected as confounders: age, BMI, SHx; social history (20)(smk; smoking, alc; alcohol, exr; exercise), PMHx; past medical history (14)(HTN; Hypertension, DM; Diabetes mellitus, DL; Dyslipidemia), and Lab; laboratory test (hsCRP (5), lnTG (34), Alb (35)). In addition, we performed multiple logistic regression to show association between serum uric acid, and abnormal BMD (at least osteopenia) and we used the same confounders mentioned above. In the subgroup analysis, we used FFM and SMI as confounders.

### 2.7 Ethics statement

The study protocol was reviewed and approved by the Institutional Review Board of the Seoul National University College of Medicine (approval no. H- 2306-037-1437).

## 3 Results

### 3.1 Baseline characteristics of study participants

The baseline characteristics of the study participants are presented in **Table 1**. Abnormal BMD was observed in 839 (28.05%) participants. Participants with abnormal BMD were older and thinner and had significantly lower serum uric acid levels (5.76 vs 6.04) than those with normal BMD. These trends were also seen in subgroup with the information on the body composition (**Supplementary Table 1** **).** In addition, the average and standard deviation of uric acid, the major variable, was 5.96 and 1.30, respectively, in primary analysis and 5.72 and 1.24, respectively, in subgroup analysis.

**Table 1:** General characteristics of participants with abnormal bone mineral density (BMD) and the control group.

Characteristics	Normal BMD (n=2,152)	Abnormal BMD (n=839)	P-value
Age (year)	59.84 ± 6.44	62.04 ± 7.12	<0.001
BMI (kg/m^2)	24.59 ± 2.72	23.22 ± 2.77	<0.001
Uric acid (mg/dL)	6.04 ± 1.31	5.76 ± 1.25	<0.001
Ca (mg/dL)	9.33 ± 0.34	9.33 ± 0.34	0.763
P (mg/dL)	3.25 ± 0.44	3.28 ± 0.46	0.141
BUN (mg/dL)	14.44 ± 3.47	14.46 ± 3.56	0.845
Cr (mg/dL)	0.96 ± 0.12	0.93 ± 0.12	<0.001
eGFR (mL/min/1.73 m^2)	82.20 ± 12.77	84.42 ± 12.97	<0.001
Alb (g/dL)	4.45 ± 0.27	4.44 ± 0.27	0.163
ALP (IU/L)	62.64 ± 16.24	66.61 ± 17.61	<0.001
FBS (mg/dL)	102.68 ± 23.98	98.37 ± 19.82	<0.001
HbA1c (%)	5.97 ± 0.80	5.85 ± 0.69	<0.001
TC (mg/dL)	196.29 ± 40.32	195.46 ± 39.35	0.608
TG (mg/dL)	123.34 ± 84.44	114.43 ± 70.01	0.007
lnTG	4.67 ± 0.52	4.61 ± 0.50	0.005
HDL (mg/dL)	51.59 ± 13.39	54.27 ± 14.79	<0.001
LDL (mg/dL)	122.93 ± 37.50	121.68 ± 37.23	0.411
OHVitD3 (ng/ml)	22.59 ± 8.57	22.64 ± 8.75	0.870
L1-4 BMD (g/cm^2)	1.30 ± 0.16	1.03 ± 0.11	<0.001
Femur neck BMD (g/cm^2)	0.99 ± 0.11	0.81 ± 0.09	<0.001
Femur total BMD (g/cm^2)	1.07 ± 0.12	0.89 ± 0.09	<0.001
Obesity (%)	871 (40.47)	202 (24.08)	<0.001
High hsCRP (%)	129 (5.99)	51 (6.08)	0.931
Smoking (smk)			
never/past (%)	1,620 (75.70)	631 (75.21)	0.780
current (%)	523 (24.30)	208 (24.79)	
Alcohol consumption (alc)			
never/past (%)	640 (29.74)	318 (37.90)	<0.001
current (%)	1,512 (70.26)	521 (62.10)	
Adequate drinker (%)	2,090 (97.12)	818 (97.50)	0.572
Adequate exercise (%)	869 (40.38)	279 (33.25)	<0.001
HTN (%)	735 (34.15)	260 (30.99)	0.099
DM (%)	393 (18.26)	101 (12.04)	<0.001
DL (%)	650 (30.20)	202 (24.08)	0.001
Osteoporosis (%)	0 (0.00)	67 (7.99)	<0.001
Osteopenia (%)	0 (0.00)	817 (97.38)	<0.001

Continuous variables are expressed as mean ± standard deviation

Categorical variables expressed as number(n) and proportion(%) of the subjects

Ca, Calcium; P, Phosphorous; BUN, Blood urea nitrogen; Cr, Creatinine; Alb, Albumin; ALP, Alkaline phosphatase; FBS, Fasting blood sugar; HbA1c, glycated hemoglobin; TC, Total cholesterol; TG, Triglyceride; HDL, High density lipoprotein; LDL, Low density lipoprotein; hsCRP, high sensitivity C-reactive protein; HTN, Hypertension; DM, Diabetes mellitus; DL, Dyslipidemia.

### 3.2 Serum uric acid level and bone mineral density

The association between serum uric acid and BMD at three sites was examined using a multiple linear regression model with the following potential confounders: age, BMI, SHx, and Lab. Positive correlations between serum uric acid level and BMD at three sites were shown in the unadjusted and fully adjusted models except for the BMD of femoral neck (P=0.054) (**Table 2**). The two-way plot showed a positive linear relationship between serum uric acid levels and BMD in the unadjusted model (**Supplementary Figure 1**).

**Table 2:** Multivariable linear regression model showing the association between serum uric acid level and bone mineral density (BMD) at three sites in the unadjusted and fully adjusted model.

	Unadjusted			Fully adjusted ^a^		
	β	SE	P-value	β	SE	P-value
L1-L4 BMD	0.016	0.003	<0.001	0.011	0.003	<0.001
FN BMD	0.011	0.002	<0.001	0.004	0.002	0.054
FT BMD	0.013	0.002	<0.001	0.005	0.002	0.004
				Alc omitted due to collinearity		

a Adjusted for age, body mass index, SHx (smk, alc, exr), PMHx (HTN, DM, DL), and Lab (hsCRP, lnTG, Alb).

β, Beta(β)-coefficients; SE, Standard error.

In subgroup analysis, we additionally adjusted for FFM and SMI in the unadjusted and fully adjusted models. All had a statistically significant positive association (p<0.05) (**Supplementary Table 2**).

### 3.3 Serum uric acid level and abnormal bone mineral density

**Table 3** shows the odds ratio (OR) of serum uric acid level for abnormal BMD using multivariable logistic regression analysis with the same confounders such as age, BMI, SHx, PMHx, and Lab. The adjusted OR of serum uric acid level for abnormal BMD was 0.91 [95% confidence interval (CI) = 0.85–0.98, P = 0.008].

**Table 3:** Crude and adjusted odds ratio [OR] (95% confidence interval [CI]) of serum uric acid level for abnormal BMD in primary analysis.

	OR (95% CI)	P-value
Unadjusted	0.84 (0.79-0.900)	<0.001
Fully adjusted ^a^	0.91 (0.85-0.98)	0.008

a Adjusted for age, body mass index, SHx (smk, alc, exr), PMHx (HTN, DM, DL), and Lab (hsCRP, lnTG, Alb).

In the subgroup analysis, we further adjusted for FFM and SMI. The adjusted OR of serum uric acid level for abnormal BMD was 0.88 (95% CI=0.79-0.997) in multivariate adjusted model, 0.88 (0.78-0.99) in the further adjusted model for FFM and 0.88 (0.78-0.995) in the further adjusted model for SMI (**Table 4**).

**Table 4:** Crude and adjusted odds ratio[OR] (95% confidence interval [CI]) of serum uric acid level for abnormal BMD in the subgroup analysis.

	OR (95% CI)	P-value
Unadjusted	0.83 (0.74-0.92)	0.001
Fully adjusted ^a^	0.88 (0.79-0.997)	0.044
Fully adjusted ^a^ with FFM	0.88 (0.78-0.99)	0.038
Fully adjusted ^a^ with SMI	0.88 (0.78-0.995)	0.041

a Adjusted for age, body mass index, SHx (smk, alc, exr), PMHx (HTN, DM, DL), and Lab (hsCRP, lnTG, Alb).

### 3.4 Fat free mass and skeletal muscle index and serum uric acid level and bone mineral density in subgroup analysis

FFM and SMI showed positive association with serum uric acid level and BMD at three sites, with statistical significance (p<0.05) (**Supplementary Tables 3**, **4** **) (** **Supplementary Figure 2**).

## 4 Discussion

In the present study, we demonstrated a positive association between serum uric acid level and BMD at three sites (lumbar spine 1-4, femoral neck, and femur total) and a protective effect against abnormal BMD after adjusting for multiple confounders, including FFM and SMI in men aged ≥ 50 years. In addition, FFM and SMI showed positive association with serum uric acid level and BMD at three sites, with statistical significance.

The main results of our study were consistent with those of a previous large multicenter Chinese study that divided uric acid level into quartiles, and as uric acid level increased from Q1 to Q4, BMD increased and the risk of abnormal BMD decreased even after adjusting for confounders in men >50 years of age and postmenopausal women (35). Besides, a systematic review and meta-analysis by Veronese N. et al. in 2016 reported that high serum uric acid level was associated with high BMD at the lumbar spine, hip joint, and femoral neck, low prevalence of osteoporosis after adjusting for confounders and low risk of new fractures in longitudinal follow-ups (8). The results of following studies were not consistent with our study findings. A US study of 6,704 participants men aged >18 years found no association between serum uric acid level and lumbar BMD after correcting for confounders (3). First, this study included young men aged <50 years and investigated only lumbar BMD (2, 30). A study involving a cross-sectional and longitudinal analyses of Korean postmenopausal women showed no association between tertiles of serum uric acid level and BMD in the spine and femoral neck. However, the sample size was small (328 and 186), and the mean interval length was relatively short (14.6 months) (36).

Oxidative stress is a state of excessive production of free radicals, reactive oxygen species (ROS) and reduced antioxidative system (6). It stimulates osteoclast differentiation and suppresses osteoblast differentiation via ERK and ERK-dependent NF-Kb activation demonstrated in experiment, resulting in bone loss (10). In addition, it promotes myofibrillar proteolysis and muscle atrophy (17, 37–39). The result of our study can be explained by the antioxidant effect of uric acid, which reduces oxidative stress-related bone loss (5, 8, 14). Furthermore, an *in vitro* study showed that administration of uric acid reduced osteoclastogenesis in a dose-dependent manner leading to reduced bone resorption (8, 40).

The subgroup analysis indicated that mechanical muscle force affects on bone strength; moreover it is affected by how much body mass is supported by the muscle and bone (16). In addition, the muscle-derived mechanical loading and cytokines via the endocrine and paracrine pathway stimulate bone development and maintenance (17, 38).

This study has several strengths. First, we excluded patients with medical histories that could affect bone metabolism. Additional laboratory tests were performed to identify patients with hyperthyroidism, carriers of hepatitis B or C and chronic kidney disease. Second, we adjusted for multiple confounders (5, 35, 36). Third, we included not only osteoporosis but also osteopenia, which has a rising prevalence and growing in importance (2, 4) and used the dual-energy X-ray absorptiometry, which is a gold standard diagnostic tool (30).

This study has several limitations. First, it was a retrospective cross-sectional study; therefore, we could not determine the causality between serum uric acid levels and BMD. Second, the participants had spontaneously comprehensive medical examination at a university hospital; therefore, this may cause a bias and the participants may have had different characteristics from the general population. Third, uric acid level was measured once as the main variable. Another previous study measured baseline uric acid levels retrospectively and used the average value (17). Fourth, fracture history was not checked.

Despite these limitations, this is the first large-scale Korean study to show that serum uric acid may have a protective effect on BMD, even after adjusting for SMI and FFM. Therefore, it is important to acknowledge the dual role of uric acid in clinical practice (5). Further longitudinal studies are needed to examine the causal association among serum uric acid levels, bone mineral density, and osteoporotic fractures.

## Data availability statement

The original contributions presented in the study are included in the article/**Supplementary Material**. Further inquiries can be directed to the corresponding author.

## Ethics statement

The studies involving humans were approved by SEOUL NATIONAL UNIVERSITY HOSPITAL BIOMEDICAL RESEARCH INSTITUTE. The studies were conducted in accordance with the local legislation and institutional requirements. The ethics committee/institutional review board waived the requirement of written informed consent for participation from the participants or the participants’ legal guardians/next of kin because This is a large scale retrospective cross-sectional study, so we can’t obtain the individual agreement. And the data was anonymized, so it is assumed to little risk to the participants.

## Author contributions

SK: Conceptualization, Data curation, Formal analysis, Investigation, Methodology, Resources, Validation, Visualization, Writing – review & editing, Writing – original draft. SL: Data curation, Formal analysis, Methodology, Resources, Writing – review & editing. HK: Conceptualization, Data curation, Formal analysis, Investigation, Methodology, Resources, Validation, Visualization, Writing – review & editing, Funding acquisition, Supervision.

## References

[1] Oh KW. Osteoporosis. Korean J Med (2008) 75(3):267–73.

[2] Kang MI Kho JM Gong HS Kwon YJ Kwon YD Kim KM. Physician’s Guide for osteoporosis : Korean society for bone and mineral research. (2022).

[3] Li X Li L Yang L Yang J Lu H. No association between serum uric acid and lumbar spine bone mineral density in US adult males: a cross sectional study. Sci Rep (2021) 11(1):15588. doi: 10.1038/s41598-021-95207-z 34341438 PMC8329127

[4] Kim JH Yoon JE Park CM Jo AJ Park KI Kong SH. A Study on the necessity of prevention treatment for fracture in osteopenia patients. Natl Evidence-Based Healthcare Collaborating Agency (2021).

[5] Gherghina M-E Peride I Tiglis M Neagu TP Niculae A Checherita IA. Uric acid and oxidative stress—Relationship with cardiovascular, metabolic, and renal impairment. Int J Mol Sci (2022) 23(6):3188. doi: 10.3390/ijms23063188 35328614 PMC8949471

[6] Pasalic D Marinkovic N Feher-Turkovic L. Uric acid as one of the important factors in multifactorial disorder-facts and controversied. Biochemia Med (2012) 22(1):63–75. doi: 10.11613/BM.2012.007 PMC4062324 22384520

[7] Joosten LA Crişan TO Bjornstad P Johnson RJ. Asymptomatic hyperuricemia: a silent activator of the innate immune system. Nat Rev Rheumatol (2020) 16(2):75–86. doi: 10.1038/s41584-019-0334-3 31822862 PMC7075706

[8] Veronese N Carraro S Bano G Trevisan C Solmi M Luchini C. Hyperuricemia protects against low bone mineral density, osteoporosis and fractures: a systematic review and meta-analysis. Eur J Clin Invest (2016) 46(11):920–30. doi: 10.1111/eci.12677 27636234

[9] De Oliveira EP Burini RC. High plasma uric acid and concentration : causes and consequences. Diabetol Metab Syndrome (2012) 4:12. doi: 10.1186/1758-5996-4-12 PMC3359272 22475652

[10] Lin K-M Lu C-L Hung K-C Wu P-C Pan C-F Wu C-J. The Paradoxical Role of Uric acid in osteoporosis. Nutrients (2019) 11(9):2111. doi: 10.3390/nu11092111 31491937 PMC6769742

[11] Zhang D Bobulescu IA Maalouf NM Adams-Huet B Poindexter J Park S. Relationship between serum uric acid and bone mineral density in the general population and in rats with experimental hyperuricemia. J Bone Mineral Res (2015) 30(6):992–9. doi: 10.1002/jbmr.2430 PMC4439277 25491196

[12] Kang S Kwon D Lee J Chung Y-J Kim M-R Namkung J. Association between serum uric acid levels and bone mineral density in postmenopausal women: A cross-sectional and longitudinal study. Healthcare (Basel). (2021) 9(12):1681. doi: 10.3390/healthcare9121681 34946407 PMC8701215

[13] Li J-Y Lee J-I Lu C-C Su Y-D Chiu C-T Chen S-C. Hyperuricemia and its association with osteoporosis in a large Asian Cohort. Nutrients. (2022) 14(11):2206. doi: 10.3390/nu14112206 35684005 PMC9182899

[14] Lee JW Kwon BC Choi HG. Analyses of the relationship between hyperuricemia and osteoporosis. Sci Rep (2021) 11(1):1–8. doi: 10.1038/s41598-021-91570-z 34103622 PMC8187414

[15] Kim MJ Sung EJ Kim CH Shin HC Lee SY. Association of lumbar spine bone mineral density according to obesity and metabolic health status in korean 60 years of age or older. Korean J Family Practice. (2018) 8(4):593–600. doi: 10.21215/kjfp.2018.8.4.593

[16] Ho-Pham LT Nguyen UD Nguyen TV. Association between lean mass, fat mass, and bone mineral density: a meta-analysis. J Clin Endocrinol Metab (2014) 99(1):30–8. doi: 10.1210/jc.2013-3190 24384013

[17] Dong XW Tian HY He J Wang C Qiu R Chen YM. Elevated serum uric acid is associated with greater bone mineral density and skeletal muscle mass in middle-aged and older adults. PloS One (2016) 11(5):e0154692. doi: 10.1371/journal.pone.0154692 27144737 PMC4856375

[18] Liu X Chen X Hu F Xia X Hou L Zhang G. Higher uric acid serum levels are associated with sarcopenia in west China: a cross-sectional study. BMC Geriatrics. (2022) 22(1):1–9. doi: 10.1186/s12877-022-02817-x 35151263 PMC8841067

[19] BeaversKMBeaversDPSerraMCBowdenRGWilsonR. Low relative skeletal muscle mass indicative of sarcopenia is associated with elevations in serum uric acid levels: findings from NHANES III. J Nutrition Health Aging. (2009) 13(3):177–82. doi: 10.1007/s12603-009-0054-5 19262948

[20] Joo NS Kim BT Gong MH Park SB Lee TY Kim KM. Impact of smoking and alcohol intake on bone mineral density in men. Korean J Family Practice. (2006) 27(11):911–6.

[21] Jung JG Kim JS Yoon SJ Lee SM Ahn SK. Korean alcohol guidelines for primary care physician. Korean J Family Practice. (2021) 11(1):14–21. doi: 10.21215/kjfp.2021.11.1.14

[22] The physical activity guide for Koreans. Ministry of health and welfare. Korean Health Promotion agency (2023) p 34–41.

[23] Hong AR Ahn HY Kim BK Ahn SH Park SY Kim MH. Evaluation and management of bone health in patients with thyroid diseases: a position statement from the korean thyroid association. Int J Thyroidology. (2022) 15:1–16. doi: 10.11106/ijt.2022.15.1.1

[24] Nah EH Park JY Kim SI. Prevalence of osteopenia in female HBV carriers and its correlation with liver function test. Ann Lab Med (2005) 3(2):181–8.

[25] Lee HJ. White Paper on Liver Diseases in Korea : Korean association for the study of the liver. (2021) p 72–145

[26] Lee YN Shin CS. Post-transplantation osteoporosis. J Korean Soc Transplant (2011) 25:239–44. doi: 10.4285/jkstn.2011.25.4.239

[27] Baek IH Yang SK Kim WH Kim YK Kim HJ Lee SH. Bone mineral density in newly diagnosed patients with inflammatory bowel disease. Korean J Gastroenterology. (2000) 35:439–47. 10.1046/j.1440-1746.2000.02154.x 10847438

[28] Keller J Kang J-H Lin H-C. Association between osteoporosis and psoriasis: results from the Longitudinal Health Insurance Database in Taiwan. Osteoporosis Int (2013) 24:1835–41. doi: 10.1007/s00198-012-2185-5 23052942

[29] Negishi-Koga T Gober H-J Sumiya E Komatsu N Okamoto K Sawa S. Immune complexes regulate bone metabolism through FcRγ signaling. Nat Commun (2015) 6(1):6637. 25824719 10.1038/ncomms7637

[30] Kim BT Lee SH. Diagnostic approach to osteoporosis: interpretation of bone density measurement. Korean J Family Practice. (2013) 3:6–15.

[31] Inbody® Available at: https://inbody.net.au/using-body-composition-analysis-to-manage-health-and-safety-at-work/ [Accessed June 28, 2023].

[32] Inbody® Available at: https://inbodyusa.com/general/770-result-sheet-interpretation/ [Accessed June 28, 2023].

[33] Kim DW Rhee H. Interpretation of estimated glomerular filtration rate. Korean J Med (2023) 98(1):45–51. doi: 10.3904/kjm.2023.98.1.45

[34] Lee HH Han MA Park J. Association between metabolic syndrome and osteoporosis in korean adults aged over 50 years old using the korea national health and nutrition examination survey, 2016-2017. J Health Inf Statistics. (2019) 44(3):245–52. doi: 10.21032/jhis.2019.44.3.245

[35] Lin X Zhao C Qin A Hong D Liu W Huang K. Association between serum uric acid and bone health in general population: a large and multicenter study. Oncotarget. (2015) 6(34):35395. doi: 10.18632/oncotarget.6173 26496032 PMC4742113

[36] Kang S Kwon D Lee J Chung Y-J Kim M-R Namkung J. Association between serum uric acid levels and bone mineral density in postmenopausal women: A cross-sectional and longitudinal study. Healthcare (Basel) (2021) 9(12):1681. doi: 10.3390/healthcare9121681 34946407 PMC8701215

[37] Iantomasi T Romagnoli C Palmini G Donati S Falsetti I Miglietta F. Oxidative stress and inflammation in osteoporosis: molecular mechanisms involved and the relationship with microRNAs. Int J Mol Sci (2023) 24(4):3772. doi: 10.3390/ijms24043772 36835184 PMC9963528

[38] Hsu CM Hsu CC Wu RW Huang CC Chen YC. Interplay between fat, muscle, bone mass, osteophytes and risk for tophaceous gout. J Investing Med (2023) 71(1):58–61. doi: 10.1136/jim-2022-002407 36316064

[39] Ibrahim WN Younes N Shi Z Abu-Madi MA. Serum uric acid level is positively associated with higher bone mineral density at multiple skeletal sites among healthy Qataris. Front Endocrinology. (2021) 12:653685. doi: 10.3389/fendo.2021.653685 PMC8044437 33868180

[40] Ahn S Lee S Kim B-J Lim K-H Bae S Kim E. Higher serum uric acid is associated with higher bone mass, lower bone turnover, and lower prevalence of vertebral fracture in healthy postmenopausal women. Osteoporosis Int (2013) 24:2961–70. doi: 10.1007/s00198-013-2377-7 23644878

