# Supplementary figures and images for: Association between serum uric acid level and bone mineral density in men more than 50 years of age
Source: Front Endocrinol (Lausanne). 2023 Nov 30;14:1259077. doi: 10.3389/fendo.2023.1259077 (PMC10720317; doi:10.3389/fendo.2023.1259077)

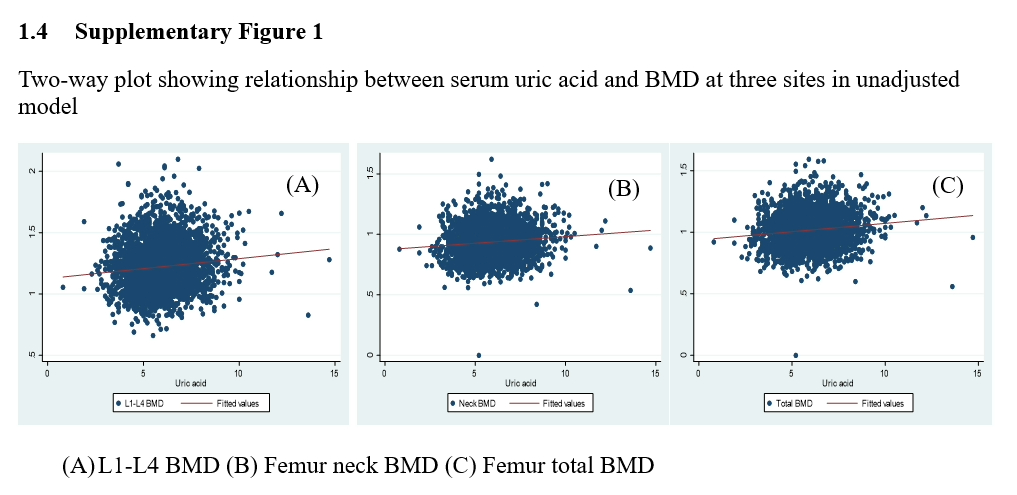

Supplement: Supplementary file 1 [file Image_1.jpg]

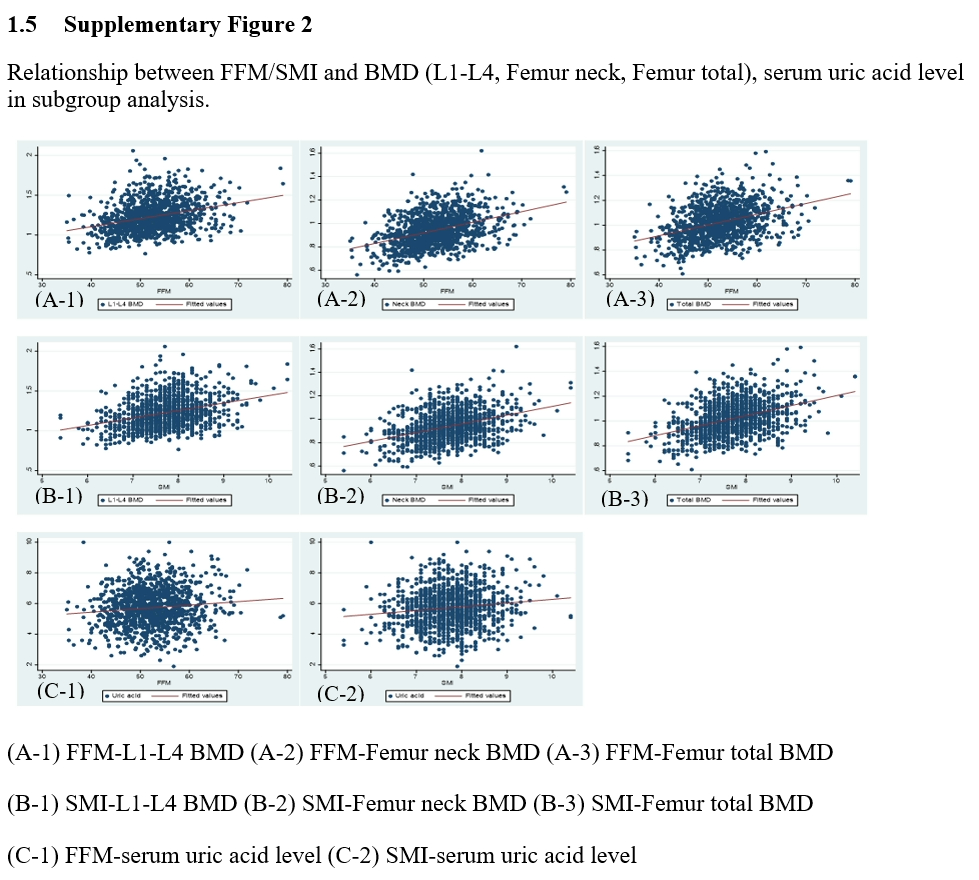

Supplement: Supplementary file 2 [file Image_2.jpeg]
