# Supplementary material for: Association between serum uric acid level and bone mineral density in men more than 50 years of age
Source: Front Endocrinol (Lausanne). 2023 Nov 30;14:1259077. doi: 10.3389/fendo.2023.1259077 (PMC10720317; doi:10.3389/fendo.2023.1259077)
Supplement: Supplementary file 3 [file Table_1.docx]

Supplementary Material

# Supplementary Tables

## Supplementary Table 1

General characteristics of the participants with information on body composition according to bone mineral density (BMD)

| **Characteristic** | **Normal BMD**  **(n=830)** | **Abnormal BMD**  **(n=305)** | **p-value** |
| --- | --- | --- | --- |
| Age (year) | 60.26 ± 6.47 | 62.67 ± 7.10 | <0.001 |
| BMI (kg/m^2) | 24.66 ± 2.78 | 23.37 ± 2.79 | <0.001 |
| BFM (kg) | 17.50 ± 5.70 | 15.46 ± 5.23 | <0.001 |
| FFM (kg) | 53.43 ± 5.67 | 49.56 ± 5.76 | <0.001 |
| SMM (kg) | 29.88 ± 3.40 | 27.52 ± 3.50 | <0.001 |
| SMI (kg/m^2) | 7.84 ± 0.63 | 7.42 ± 0.68 | <0.001 |
| Uric acid (mg/dL) | 5.80 ± 1.25 | 5.51 ± 1.20 | <0.001 |
| Ca (mg/dL) | 9.30 ± 0.35 | 9.28 ± 0.35 | 0.424 |
| P (mg/dL) | 3.21 ± 0.44 | 3.22 ± 0.45 | 0.642 |
| BUN (mg/dL) |  |  |  |
| Cr (mg/dL) | 0.95 ± 0.13 | 0.92 ± 0.12 | <0.001 |
| eGFR (mL/min/1.73 m^2) | 87.95 ± 14.20 | 90.40 ± 13.10 | 0.009 |
| Alb (g/dL) | 4.46 ± 0.28 | 4.43 ± 0.29 | 0.22 |
| ALP (IU/L) | 62.89 ± 16.10 | 66.38 ± 17.62 | 0.002 |
| FBS (mg/dL) | 103.83 ± 24.75 | 98.97 ± 20.49 | 0.002 |
| HbA1c (%) | 6.05 ± 0.82 | 5.97 ± 0.82 | 0.114 |
| TC (mg/dL) | 191.85 ± 40.66 | 192.84 ± 38.30 | 0.71 |
| TG (mg/dL) | 119.25 ± 65.41 | 112.77 ± 61.58 | 0.133 |
| lnTG | 4.66 ± 0.49 | 4.61 ± 0.47 | 0.126 |
| HDL (mg/dL) | 50.77 ± 12.97 | 53.37 ± 13.72 | 0.003 |
| LDL (mg/dL) | 120.91 ± 38.42 | 120.25 ± 36.46 | 0.79 |
| OHVitD3 (ng/ml) | 21.51 ± 8.87 | 21.36 ± 9.41 | 0.806 |
| L1-4 BMD (g/cm^2) | 1.30 ± 0.17 | 1.04 ± 0.11 | <0.001 |
| Femur neck BMD (g/cm^2) | 0.99 ± 0.11 | 0.81 ± 0.09 | <0.001 |
| Femur total BMD (g/cm^2) | 1.07 ± 0.12 | 0.89 ± 0.09 | <0.001 |
| Obesity (%) | 335 (40.36) | 76 (24.92) | <0.001 |
| High hsCRP (%) | 47 (5.66) | 13 (4.26) | 0.350 |
| Smoking (smk) |  |  |  |
| non/past (%) | 648 (78.07) | 237 (77.70) | 0.895 |
| current (%) | 182 (21.93) | 68 (22.30) |  |
| Alcohol consumption (alc) |  |  |  |
| non/past (%) | 290 (36.02) | 142 (46.56) | 0.001 |
| current (%) | 531 (63.98) | 163 (53.44) |  |
| Adequate drinker (%) | 801 (96.51) | 296 (97.05) | 0.652 |
| Adequate exercise (%) | 312 (37.59) | 82 (26.89) | 0.001 |
| HTN (%) | 292 (35.18) | 80 (26.23) | 0.004 |
| DM (%) | 160 (19.28) | 34 (11.15) | 0.001 |
| DL (%) | 263 (31.69) | 75 (24.59) | 0.020 |
| Osteoporosis (%) | 0 (0.00) | 19 (6.23) | <0.001 |
| Osteopenia (%) | 0 (0.00) | 300 (98.36) | <0.001 |

Continuous variables are expressed as mean ± standard deviation
Categorical variables expressed as number(n) and proportion(%) of the subjects
BFM, Body fat mass; FFM, Fat free mass; SMM, Skeletal muscle mass; SMI, Skeletal muscle mass index; Ca, Calcium; P, Phosphorous; BUN, Blood urea nitrogen; Cr, Creatinine; Alb, Albumin; ALP, Alkaline phosphatase; FBS, Fasting blood sugar; HbA1c, glycated hemoglobin; TC, Total cholesterol; TG, Triglyceride; HDL, High density lipoprotein; LDL, Low density lipoprotein; hsCRP, high sensitivity C-reactive protein; HTN, Hypertension; DM, Diabetes mellitus; DL, Dyslipidemia.

## Supplementary Table 2

## Association between serum uric acid level and bone mineral density (BMD) at three sites with additionally adjusted for FFM and SMI in unadjusted and fully adjusted models

|  | Unadjusted | | | Adjusted with FFM | | | Adjusted with SMI | | |
| --- | --- | --- | --- | --- | --- | --- | --- | --- | --- |
|  | L1-L4 | FN | FT | L1-L4 | FN | FT | L1-L4 | FN | FT |
| β | 0.016 | 0.014 | 0.017 | 0.011 | 0.009 | 0.012 | 0.010 | 0.008 | 0.011 |
| SE | 0.005 | 0.003 | 0.003 | 0.004 | 0.003 | 0.003 | 0.004 | 0.003 | 0.003 |
| P-value | <0.001 | <0.001 | <0.001 | 0.012 | 0.002 | <0.001 | 0.027 | 0.005 | <0.001 |
| AR^2^ | 0.010 | 0.015 | 0.021 | 0.100 | 0.166 | 0.150 | 0.109 | 0.143 | 0.162 |

|  | Fully adjusted ^a^ | | | Fully adjusted ^a^ with FFM | | | Fully adjusted ^a^ with SMI | | |
| --- | --- | --- | --- | --- | --- | --- | --- | --- | --- |
|  | L1-L4 | FN | FT | L1-L4 | FN | FT | L1-L4 | FN | FT |
| β | 0.012 | 0.006 | 0.009 | 0.012 | 0.007 | 0.009 | 0.012 | 0.007 | 0.009 |
| SE | 0.005 | 0.003 | 0.003 | 0.005 | 0.003 | 0.003 | 0.005 | 0.003 | 0.003 |
| P-value | 0.012 | 0.045 | 0.006 | 0.008 | 0.028 | 0.004 | 0.009 | 0.034 | 0.004 |
| AR^2^ | 0.092 | 0.121 | 0.138 | 0.133 | 0.189 | 0.179 | 0.134 | 0.171 | 0.183 |

^a^ Adjusted for age, body mass index, SHx (smk, alc, exr), PMHx (HTN, DM, DL), Lab (hsCRP, lnTG, Alb).
β, Beta(β)-coefficients; SE, Standard error; AR^2^, Adjusted R squared.

## Supplementary Table 3

Relationship between FFM and bone mineral density (BMD) at three sites and serum uric acid level in subgroup analysis

| FFM | L1-L4 | FN | FT | UA |
| --- | --- | --- | --- | --- |
| β | 0.010 | 0.009 | 0.009 | 0.023 |
| SE | 0.001 | 0.001 | 0.001 | 0.006 |
| AR^2^ | 0.095 | 0.160 | 0.139 | 0.011 |
| P-value | <0.001 | <0.001 | <0.001 | <0.001 |

FFM, fat free mass; FN, Femoral neck; FT, Femur total; UA, Uric acid.

β, Beta(β)-coefficients; SE, Standard error; AR^2^, Adjusted R squared.

## Supplementary Table 4

Relationship between SMI and bone mineral density (BMD) at three sites and serum uric acid level in subgroup analysis

| SMI | L1-L4 | FN | FT | UA |
| --- | --- | --- | --- | --- |
| β | 0.094 | 0.075 | 0.081 | 0.244 |
| SE | 0.008 | 0.006 | 0.006 | 0.055 |
| AR^2^ | 0.106 | 0.138 | 0.153 | 0.016 |
| P-value | <0.001 | <0.001 | <0.001 | <0.001 |

SMI, skeletal muscle mass index; FN, Femoral neck; FT, Femur total; UA, Uric acid.

β, Beta(β)-coefficients; SE, Standard error; AR^2,^ Adjusted R squared.
